# Advancements in the application of multimodal monitoring and machine learning for the development of personalized therapeutic strategies in traumatic brain injury

**DOI:** 10.3389/fnhum.2025.1695336

**Published:** 2025-10-23

**Authors:** Zhijing Wei, Lingda Meng, Wei Chong

**Affiliations:** ^1^Department of Trauma Center, First Affiliated Hospital, China Medical University, Shenyang, China; ^2^Department of Cardiac Surgery, First Affiliated Hospital, China Medical University, Shenyang, China; ^3^Department of Emergency, The First Hospital of China Medical University, Shenyang, China

**Keywords:** traumatic brain injury, multimodal monitoring, machine learning, hypothermic neuroprotection, diagnosis, prognosis

## Abstract

Trauma is the fourth leading cause of death globally and the primary cause of mortality in the 15–45 age group, with traumatic brain injury (TBI) at the core of trauma care. Annually, over 50 million TBI patients are reported worldwide. The complex and heterogeneous pathophysiology of TBI presents substantial diagnostic and therapeutic challenges. In recent years, multimodal monitoring has emerged as a crucial tool to guide clinical management. The integration of multimodal monitoring with machine learning offers novel opportunities for TBI assessment and management, given the rapid development and widespread application of machine learning approaches. Therapeutic hypothermia has shown potential neuroprotective benefits in experimental and clinical contexts, though evidence remains mixed and its implementation in practice faces significant challenges. This review summarizes recent advancements in multimodal monitoring and explores how machine learning can optimize the application of therapeutic hypothermia in conjunction with multimodal data. For example, predictive models trained on multimodal signals (e.g., EEG, ICP, cerebral blood flow, and oxygenation) can help identify patient subgroups most likely to benefit from targeted temperature management. By enabling such stratification and adaptive treatment strategies, machine learning may support the development of more personalized and effective therapeutic approaches for TBI.

## 1 Introduction

Traumatic brain injury (TBI) is a severe public health issue, affecting 10’s of millions of patients globally each year (Dewan et al., 2019; Maas et al., 2022). TBI is typically caused by external force impacting the head or penetrating injuries, potentially leading to a series of complex neurological pathological changes, including primary and secondary injuries (Wu et al., 2022). Primary injuries refer to brain tissue damage caused directly by mechanical forces, such as contusions and hemorrhages, while secondary injuries involve a series of biochemical and physiological cascades, such as brain edema, blood-brain barrier damage, inflammatory responses, oxidative stress, and apoptosis (Minta et al., 2020; Shao et al., 2019; Unadkat et al., 2025). These pathological processes often interact, leading to more extensive neuronal damage and functional impairment (Zhao et al., 2019). Clinical manifestations of TBI are diverse, including consciousness disturbances, motor dysfunction, cognitive deficits, and changes in mood and behavior (Yen et al., 2018; Zhang and Ioachimescu, 2025). Due to the complexity of TBI pathophysiological mechanisms, traditional treatment strategies often face multiple challenges. For example, single drug treatments struggle to address multiple concurrent pathological mechanisms (Lins et al., 2023), and while surgical interventions can quickly address intracranial hematomas and skull fractures, they cannot resolve microscopic neuronal damage. Moreover, individual differences result in varying effects of the same treatment plan among different patients, further increasing the difficulty of TBI treatment (Tenovuo et al., 2021; Zheng et al., 2020).

In recent years, hypothermic neuroprotection has gained widespread attention as a treatment strategy in TBI management. Hypothermic therapy reduces brain metabolic demands by lowering body temperature, thereby alleviating inflammatory responses and oxidative stress, and protecting brain tissue to some extent (Liang et al., 2023; Yan et al., 2022). However, implementing hypothermic therapy requires precise control, including cooling extent, duration, and initiation timing, to achieve optimal therapeutic effects and minimize potential side effects (Kendall et al., 2023). Despite the positive effects shown in clinical and experimental studies, standardization and personalized implementation of hypothermic neuroprotection still require further exploration (Wang et al., 2024; Wu et al., 2021).

Meanwhile, with continuous advancements in medical technology, machine learning techniques have increasingly been applied in the medical field (Greener et al., 2022). Machine learning analyzes and integrates large amounts of complex physiological data, extracting valuable feature information to support clinical decision-making (Cobianchi et al., 2023; Zhang et al., 2021). In TBI management, machine learning can improve diagnostic accuracy and treatment effectiveness by analyzing multimodal monitoring data to identify key physiological and pathological features (Bischof and Cross, 2023; Schroder et al., 2021). Thus, machine learning can monitor and analyze patients’ physiological states in real-time, providing personalized treatment recommendations and optimizing therapeutic strategies like hypothermic neuroprotection.

Multimodal monitoring technologies play a crucial role in the diagnosis and management of TBI (Roldán et al., 2020). These technologies include electroencephalography (EEG), cerebral blood flow monitoring, intracranial pressure (ICP) monitoring, imaging (such as CT and MRI), and brain oxygen saturation monitoring. Integrating various physiological parameters provides a comprehensive assessment of the brain’s condition (Rohaut et al., 2024). However, the large and complex data volumes make it challenging to efficiently extract key information using traditional methods. The introduction of machine learning offers new tools for analyzing and integrating multimodal monitoring data, enhancing the precision and personalization of TBI diagnosis and treatment (Acosta et al., 2022).

This review adopts a narrative synthesis approach rather than a systematic review method, focusing on the application of multimodal monitoring and machine learning in the management of traumatic brain injury (TBI), with particular emphasis on the integration of therapeutic hypothermia (TTM) and machine learning for personalized treatment strategies. The search strategy involved a comprehensive literature review conducted across PubMed, Scopus, and IEEE Xplore in July 2024, with studies published from January 2000 to June 2025. Key search terms included “Traumatic Brain Injury (TBI),” “Multimodal monitoring,” “Machine learning,” “Hypothermic neuroprotection,” “Therapeutic Hypothermia (TTM),” “Electroencephalography (EEG),” “Cerebral Blood Flow (CBF),” “Intracranial Pressure (ICP),” “Brain Oxygen Saturation (PbtO2)”, and “Clinical Decision Support.” Studies were included if they discussed the application of multimodal monitoring or machine learning in TBI diagnosis, treatment, or management, and those focused on therapeutic hypothermia or the integration of multiple data modalities with machine learning for personalized care. Exclusion criteria comprised articles not addressing TBI or multimodal monitoring, studies limited to animal models, and publications outside the defined time range (before 2000 or after June 2025). The studies were grouped based on monitoring modality (e.g., EEG, CBF, ICP, PbtO2, CT/MRI imaging), task (e.g., diagnosis, prognosis, prediction, therapeutic intervention), and outcome (e.g., effectiveness of monitoring methods, clinical outcomes, machine learning model performance). This review does not perform a formal systematic quality assessment, instead providing a qualitative synthesis of key themes and trends. While areas of conflicting or insufficient evidence were noted, especially concerning the integration of machine learning with therapeutic hypothermia, the methodology remains transparent and reproducible, ensuring that the review’s findings are grounded in the existing literature. By clearly defining inclusion and exclusion criteria, the review enables a comprehensive analysis of multimodal monitoring and machine learning in TBI, offering valuable insights into current practices and highlighting areas for further research and innovation.

This article will delve into the application of hypothermic neuroprotection in TBI management and analyze the role of machine learning in optimizing hypothermic therapy. Additionally, it will review the importance of multimodal monitoring technologies in TBI assessment and how machine learning improves the effectiveness and personalization of TBI treatment through data analysis. Future research will explore advancing personalized and precise TBI management based on these emerging technologies. This review is intended for neurocritical care clinicians, biomedical engineers, and researchers focused on traumatic brain injury (TBI) management and treatment innovation. Its primary aim is to provide decision-support insights for healthcare professionals involved in the acute management of TBI, particularly in the application of multimodal monitoring and machine learning for personalized treatment strategies.

## 2 Multimodal monitoring technologies

**FIGURE 1:**
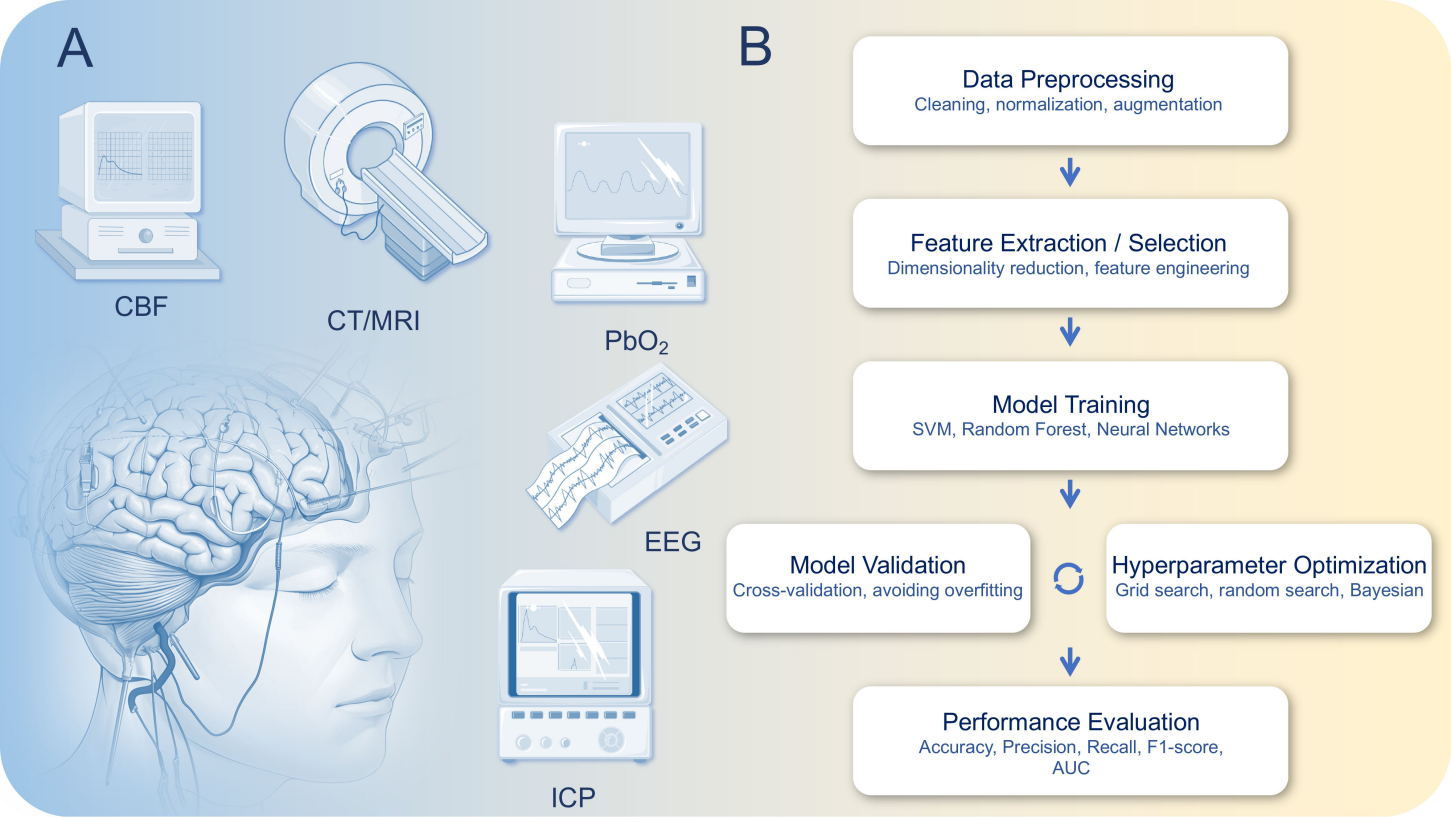
**(A)** Multimodal monitoring technology for traumatic brain injury (TBI) and **(B)** typical workflow of a machine learning algorithm, illustrating the main steps including data preprocessing, feature extraction/selection, model training, model validation, hyperparameter optimization, and performance evaluation. This pipeline ensures robust and generalizable model performance for biomedical data analysis.

Multimodal monitoring technologies provide clinicians with rich information sources to more comprehensively and accurately assess patients’ neurological states (Appavu et al., 2021; Hwang et al., 2025; Tas et al., 2022). As shown in Figure 1A, the key components of multimodal monitoring are EEG, Cerebral Blood Flow Monitoring, ICP Monitoring, Imaging, Brain Oxygen Saturation Monitoring, etc., Figure 1B shows the typical workflow of a typical machine learning algorithm. The following Table 1 summarizes the key technical parameters and applications of various monitoring techniques used during different phases of Traumatic Brain Injury (TBI). It highlights the core technical features of each technology, such as sampling frequency, spatial resolution, and monitoring depth. In the acute phase, these technologies focus on quick diagnosis and timely intervention, with an emphasis on monitoring brain function, detecting ischemic events, and identifying high intracranial pressure (ICP). In the subacute phase, the technologies are used to assess treatment efficacy and monitor brain recovery. Finally, in the recovery phase, these tools help evaluate long-term outcomes and support decisions regarding rehabilitation strategies. This table provides a comprehensive overview of how each technology is applied in TBI management and recovery, offering insights into their role and effectiveness across different stages of the injury.

**TABLE 1:** Technical parameters and phase-specific applications of monitoring technologies in traumatic brain injury (TBI).

Technology	Core parameters	Technical features	Acute phase application	Subacute phase application	Recovery phase application
EEG	Sampling frequency: 250–500 Hz	Records electrical activity from the brain’s cortex, reflecting brain function	Monitors seizures, consciousness changes, and brain dysfunction in real-time	Assesses brain activity recovery and aids in post-injury brain function evaluation	Long-term monitoring of brain electrical activity for recovery assessment
	Spatial resolution: 2–3 cm	Can be combined with machine learning for improved diagnostic accuracy	Helps identify abnormal brain waves for early intervention	Monitors brain electrical activity recovery, aids in developing recovery plans	Long-term monitoring to evaluate recovery outcomes
Laser doppler flowmetry	Spatial resolution: 0.5–2 mm	Assesses local cerebral blood flow, detects ischemia and hypoxia	Quickly diagnoses ischemic events, evaluates brain perfusion status	Assesses treatment effects, evaluates blood flow changes	Monitors brain blood flow recovery and evaluates perfusion restoration
	Sampling frequency: 1–10 Hz	Suitable for dynamic brain blood flow monitoring	Identifies ischemic events for clinical intervention	Evaluates treatment effects, optimizes therapeutic strategies	Long-term monitoring of blood flow recovery in the brain
NIRS	Sampling frequency: 1–10 Hz	Measures brain tissue oxygenation, provides continuous monitoring of cerebral oxygen saturation	Real-time monitoring of oxygen saturation, identifies hypoxia and low perfusion	Assesses oxygenation status, helps adjust therapeutic strategies	Continuous monitoring of brain oxygenation status during recovery
	Depth: 2–3 cm	Provides oxygenation status for shallow brain tissue	Helps guide treatment of hypoxic events	Assesses oxygenation recovery, monitors the effectiveness of hypoxic treatments	Evaluates long-term oxygenation status during recovery
ICP monitoring	Sampling frequency: 1–10 Hz	Assesses intracranial pressure, evaluates brain edema and hypertension	Monitors ICP fluctuation, timely intervention in high ICP situations	Assesses edema changes, evaluates treatment effects	Long-term ICP monitoring to assess brain edema and recovery status
	Non-invasive monitoring (acoustic methods)	Quick, non-invasive method for ICP assessment	Provides real-time ICP data for urgent clinical decisions	Monitors ICP and edema for treatment adjustments	Monitors ICP during recovery, aiding in later treatment decisions
CT	Resolution: 0.5–1 mm	Rapid imaging for intracranial hemorrhages, fractures, and edema	Quickly identifies brain hemorrhages and fractures in emergency settings	Assesses diffuse injuries, brain tissue damage	Evaluates recovery of brain injuries, guides post-treatment decisions
	Scan time: seconds to a few minutes	Fast imaging for urgent diagnosis	Used for emergency screening in acute phase to avoid missed fatal injuries	Helps assess chronic damage, assists in treatment decisions	Provides follow-up imaging to assess recovery progress
MRI	Resolution: 1 mm	High-resolution imaging of soft brain tissues, assesses deep brain injuries	Identifies diffuse axonal injury, parenchymal changes in acute phase	Provides detailed imaging of soft tissue damage, assesses chronic injury recovery	Long-term imaging to assess white matter damage and evaluate recovery outcomes
	Scan time: 10–30 min	Ideal for assessing soft brain tissue injuries in subacute and recovery phases	Suitable for subacute phase to evaluate white matter injury and guide treatment	Evaluates soft tissue recovery, determines therapeutic strategy	Long-term assessment of white matter damage and neurological recovery

### 2.1 EEG

Electroencephalography records electrical activity from the brain’s cortex, providing a non-invasive and real-time method for assessing brain function. In TBI patients, EEG can monitor phenomena such as seizures, consciousness changes, and brain dysfunction. Common EEG technical parameters include: Sampling Frequency: Typically, between 250 and 500 Hz, used to capture high-frequency cortical activity. Spatial Resolution: Generally, 2–3 cm, used to localize electrical activity in the cerebral cortex.

Patients with TBI often face the risk of seizures; EEG helps in the timely identification and monitoring of seizure activities for early intervention (Pyrzowski et al., 2024; Sconzo et al., 2025). Additionally, EEG is used to evaluate consciousness levels and the extent of brain dysfunction, aiding clinical decision-making (Bai et al., 2021). However, traditional EEG data interpretation relies on experienced neurologists and is susceptible to noise. Combining machine learning technology allows for automated analysis of EEG data, identifying specific brain wave patterns and improving analysis efficiency and accuracy (Parsa et al., 2023). For example, machine learning algorithms can quickly detect abnormal brain activities and assist clinicians in making more accurate diagnoses, using deep learning to automatically detect epileptiform abnormalities in EEG from TBI. They demonstrated that a recurrent neural network (RNN) trained with continuous electroencephalogram (EEG) data can effectively identify epileptiform activity (EA), achieving an accuracy of up to 80.78%. This lays the foundation for robust and automated detection of epileptiform activity in traumatic brain injury (TBI) patients (Faghihpirayesh et al., 2021).

In the acute phase of TBI, EEG is primarily used to monitor epileptic activity for early detection and intervention. During the subacute phase, EEG can assess the recovery of brain function. In the recovery phase, EEG is used to evaluate long-term neurological recovery. Machine learning techniques can analyze abnormal waveforms in EEG signals, automatically detect seizures, and assist clinical diagnosis, thus enhancing the efficiency of analysis.

### 2.2 Cerebral blood flow monitoring

Cerebral blood flow monitoring assesses the brain’s blood perfusion status, providing crucial information about ischemia and hypoxia. The technical parameters are as follows: Laser Doppler Flowmetry: Spatial Resolution: Approximately 0.5–2 mm, used to monitor dynamic changes in local cerebral blood flow. Sampling Frequency: Typically 1–10 Hz, used for real-time blood flow fluctuation assessment. NIRS: Oxygen Saturation Detection Range: Usually between 60% and 100%. Depth: NIRS is generally used for monitoring oxygenation status of superficial brain tissue, with a depth of 2–3 cm.

The stability of cerebral blood flow is vital for maintaining normal brain function (Vu et al., 2024) In TBI, cerebral blood flow monitoring can be achieved through techniques like laser Doppler flowmetry, thermal diffusion, and near-infrared spectroscopy. Laser Doppler flowmetry evaluates local brain blood flow changes by measuring laser reflection (Ayasse et al., 2025), while thermal diffusion uses thermal probes to measure temperature changes in local tissues, indirectly reflecting blood flow (Hartings et al., 2020). Near-infrared spectroscopy measures cerebral oxygen saturation, providing continuous and non-invasive information on central nervous system hemoglobin oxygen saturation (Barud et al., 2021). These technologies play a crucial role in identifying ischemic events and assessing treatment effects. Cerebral blood flow data are often complex and dynamic, and machine learning can more accurately interpret these data through pattern recognition and trend analysis, providing decision support. By analyzing real-time cerebral blood flow data, machine learning can identify potential ischemic risks and offer recommendations for clinical intervention.

In the acute phase of TBI, laser Doppler flowmetry helps identify cerebral ischemic events. During the subacute phase, NIRS can continuously monitor the oxygenation status of brain tissue. In the recovery phase, these techniques assist in evaluating cerebral blood flow recovery.

### 2.3 ICP monitoring

Intracranial pressure monitoring is a key tool for evaluating intracranial hypertension and brain edema (Shang et al., 2024). Elevated ICP is a common complication in TBI, potentially leading to insufficient brain perfusion and neuronal damage. Common monitoring methods include external sensors and non-invasive techniques. Key parameters include: Sampling Frequency: Typically, 1–10 Hz, used for real-time ICP fluctuation monitoring. Non-invasive ICP Monitoring: ICP changes are estimated using cranial acoustic techniques, suitable for initial assessments in acute phase patients.

Intracranial pressure monitoring is typically performed using external sensors and non-invasive techniques. External sensors measure ICP directly through intracranial pressure sensors, while non-invasive monitoring estimates ICP changes using cranial acoustic techniques. Real-time monitoring of ICP changes provides a basis for managing intracranial hypertension and aids in determining whether surgical intervention or treatment adjustments are necessary (Fernando et al., 2019). However, due to the dynamic nature of ICP data and individual variations, personalized solutions are required (Zeiler et al., 2022). Machine learning can help identify potential crisis moments through big data analysis and provide personalized management plans. Machine learning models can also predict ICP trends, helping doctors take preventive measures in advance.

In the acute phase, ICP monitoring is used to identify elevated intracranial pressure early and guide treatment. During the subacute phase, ICP monitoring helps assess changes in brain edema, and in the recovery phase, it is used to evaluate ICP recovery and guide long-term management.

### 2.4 Imaging

Imaging is a fundamental method for TBI assessment, providing structural and functional information (Hu et al., 2022). Common imaging techniques include computed tomography (CT) (Mader et al., 2021), magnetic resonance imaging (MRI) (Pinggera et al., 2020), and proton magnetic resonance spectroscopy (Bartnik-Olson et al., 2021). Technical parameters include: CT: Resolution: Typically, 0.5–1 mm, used for rapid identification of intracranial hemorrhage and fractures. Scan Time: The rapidity of CT scanning makes it the method of choice during the acute phase. MRI: Resolution: Typically, 1 mm, used for assessing soft brain tissue damage, diffuse axonal injury, and white matter lesions.

Computed tomography scans quickly identify intracranial hemorrhages, fractures, and edema, making it the preferred imaging method in emergency situations, while MRI offers higher-resolution imaging of brain soft tissues, detecting diffuse axonal injury and parenchymal changes. Imaging provides precise localization of lesions, assesses the extent and nature of damage, and forms the basis for personalized treatment plans. However, interpreting imaging data requires expertise, and machine learning can enhance analysis efficiency through automated image recognition and segmentation. Machine learning algorithms can automatically identify lesion areas in CT or MRI scans, assisting doctors in more accurate evaluations (Ling et al., 2025).

In the acute phase, CT is used for rapid screening of hemorrhage and fractures. In the subacute phase, MRI helps assess diffuse injuries and white matter changes, with advantages in evaluating soft brain tissues. During the recovery phase, MRI is used to detect neural repair and functional brain recovery.

### 2.5 Brain oxygen saturation monitoring

Brain oxygen saturation monitoring assesses the oxygenation status of brain tissue, providing critical information for identifying hypoxia and low perfusion. Technical parameters include: Oxygen Saturation Detection Range: Typically, between 60% and 100%, reflecting the oxidative status of brain tissue in real time. Monitoring Depth: NIRS is typically used for monitoring superficial brain tissue with a depth of 2–3 cm.

Near-infrared spectroscopy (NIRS) is a commonly used technique for monitoring brain oxygen saturation (Gomez et al., 2023a). This technology evaluates oxygenation status by measuring changes in tissue light absorption, enabling real-time monitoring of brain oxygen saturation, identifying potential hypoxic events, and assessing the impact of treatment on brain oxygenation. An article from the Canadian High-Resolution Traumatic Brain Injury (CAHR-TBI) cohort study explored the predictive value of regional oxygen saturation (rSO_2_) and cerebrovascular reactivity index (CVRi) measured by near-infrared spectroscopy in acute traumatic brain injury patients’ prognosis. The study found that these two indicators were significantly correlated with patients’ clinical outcomes, with low rSO_2_ and CVRi associated with poor prognosis, suggesting that NIRS may become an effective tool for assessing acute TBI prognosis and guiding clinical treatment decisions (Gomez et al., 2024). Brain oxygen saturation data are easily influenced by external environment and physiological factors. Machine learning can improve data accuracy through data cleaning and feature extraction, help identify potential oxygenation issues, and detect key features related to prognosis, thus guiding personalized treatment (Gomez et al., 2023b).

In the acute phase of TBI, NIRS is used for continuous monitoring of brain tissue oxygenation. During the subacute phase, combined with other multimodal data, NIRS helps evaluate brain hypoxia and guide treatment. In the recovery phase, this technology assists in evaluating the oxygenation status during the process of neurological recovery.

In addition to non-invasive modalities such as NIRS, invasive brain tissue oxygenation techniques [e.g., LICOX (Früh et al., 2024), Raumedic (Rot et al., 2020)] remain the standard in many centers, and recent trials such as BOOST and BONANZA are exploring their role in TBI management.

## 3 Application of machine learning in TBI

**FIGURE 2:**
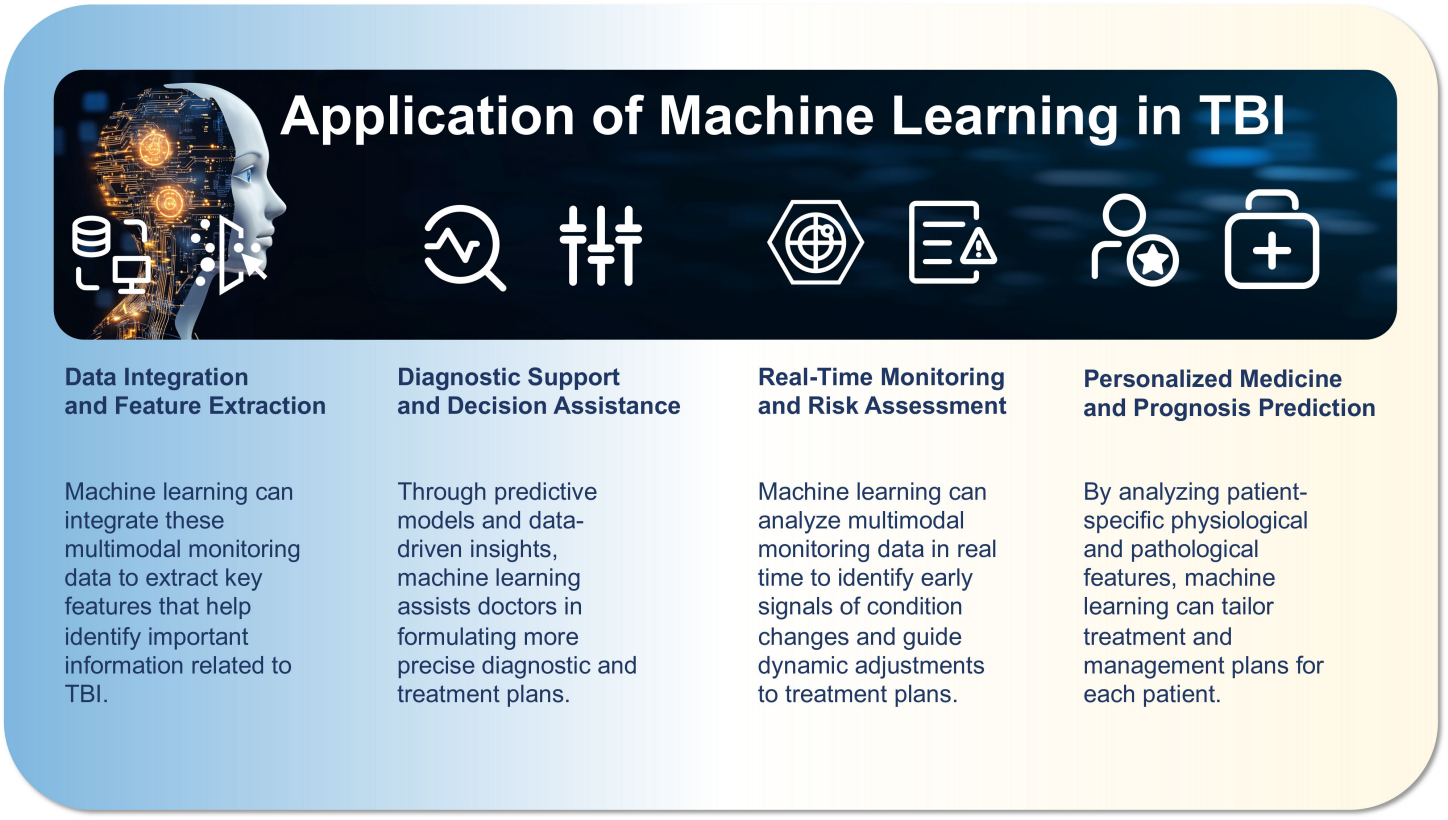
Application of machine learning in traumatic brain injury (TBI).

The application of machine learning in TBI management is increasingly widespread, demonstrating significant potential. The success of machine learning models in diagnosing and assessing TBI relies heavily on efficient feature extraction. To achieve this, both traditional statistical methods, such as Principal Component Analysis (PCA) and Independent Component Analysis (ICA), and deep learning approaches, such as Convolutional Neural Networks (CNN), are employed to extract key information from multimodal data, including EEG, CT, and MRI. In the training and validation process, the dataset is typically split into training and validation sets at ratios of 70%–30% or 80%–20%, with cross-validation occasionally used to further assess the model’s generalization capability. During model training, hyperparameters are optimized through techniques like grid search or random search to enhance model accuracy and stability. For model validation and performance evaluation, a test set is used, and performance is assessed through tools such as AUC-ROC curves and confusion matrices. In addition to standard metrics like accuracy, precision, and recall, supplementary indicators such as F1-score and Mean Squared Error (MSE) are used for a more comprehensive evaluation. Common algorithms used in this context include CNNs, which automatically extract spatial features from EEG data to aid in TBI diagnosis, and Support Vector Machines (SVM), which are effective for classifying small, high-dimensional datasets, often used to distinguish between mild and severe TBI. Through automated analysis and data-driven insights, machine learning is transforming TBI diagnosis and treatment, especially in the integration of multimodal monitoring data, development of personalized treatment plans, and optimization of treatment strategies (as shown in Figure 2).

### 3.1 Data integration and feature extraction

Traumatic brain injury patients often undergo multiple monitoring techniques, generating large amounts of complex physiological data. Machine learning can integrate these multimodal monitoring data to extract key features that help identify important information related to TBI. For example, extracting brain activity patterns from EEG, detecting lesion areas from imaging data, and identifying ischemic states from cerebral blood flow and oxygen saturation data. This feature extraction capability not only improves diagnostic accuracy but also provides strong support for developing personalized treatment plans.

EEG data analysis: Machine learning can automate the analysis of EEG signals to identify specific brain activity patterns, such as warning signals for seizures. This helps in early intervention before the patient shows obvious symptoms. For instance, Vishwanath et al. (2021), explored the possibility of detecting mild traumatic brain injury (mTBI) through machine learning and deep learning techniques applied to human sleep EEG data. The study found that these algorithms effectively identified mTBI patients from EEG data, showing high accuracy and sensitivity.

Imaging data processing: In CT and MRI imaging data, deep learning algorithms can automatically identify and segment lesion areas, reducing the burden on doctors and increasing diagnostic efficiency. For example, Muller et al. (2023) successfully developed a model for classifying chronic TBI using machine learning techniques on mixed diffusion imaging data. This study revealed the potential of machine learning to enhance the accuracy of chronic TBI diagnosis, especially in identifying damage patterns and differentiating stages of injury. This work provides clinicians with a data-driven tool to improve the diagnosis and treatment planning for chronic TBI patients (Muller et al., 2023).

Multisource data integration: By integrating data from different monitoring technologies, machine learning can generate a comprehensive patient status model, enabling more accurate disease assessment and prognosis prediction.

The following Table 2 provides a comparison of various monitoring modalities used in TBI management, detailing their respective data sources, prediction or diagnosis tasks, model types, validation methods, performance metrics, and limitations. It summarizes the application of different machine learning models across diverse modalities such as EEG, ICP, CBF, PbtO2, and imaging (CT/MRI), highlighting key aspects like the model class, primary metrics (e.g., AUC, accuracy, sensitivity), and potential limitations. By examining these parameters, the table offers insights into the strengths and challenges associated with each monitoring modality, aiding in the selection of the most appropriate method for personalized TBI management.

**TABLE 2:** Comparison of monitoring modalities, models, and performance metrics in traumatic brain injury (TBI).

Monitoring modality	Prediction/diagnosis task	Model class	Validation type	Primary metrics	Calibration/decision curve	Key limitations
EEG	Seizure detection, consciousness level prediction	Deep learning (CNN, RNN)	Internal cross-validation	AUC, accuracy, sensitivity	Decision curve analysis available	Noise sensitivity, dependence on expert interpretation
ICP	ICP prediction	Random forest	External multi-site validation	RMSE, AUC, MAE	Calibration curves reported	Artifact handling, missing data
CBF	Ischemia detection, blood flow dynamics	SVM, decision trees	Internal CV, temporal split	AUC, sensitivity, specificity	No decision-curve analysis	Limited by imaging resolution, small sample size
PbtO2	Oxygenation monitoring, prognosis	Logistic regression	Internal CV, external validation	AUC, sensitivity, PPV	Decision curve not included	Calibration issues, poor external validation
Imaging (CT/MRI)	Lesion detection, injury severity classification	CNN, U-Net	Internal CV, external validation	AUC, dice coefficient, IoU	Calibration curve not reported	Limited by imaging quality, time-consuming interpretation

### 3.2 Diagnostic support and decision assistance

Machine learning applications in TBI extend beyond data analysis to provide substantial support for clinical decision-making. Through predictive models and data-driven insights, machine learning assists doctors in formulating more precise diagnostic and treatment plans.

Diagnostic support systems: Based on multimodal data, machine learning models can not only automate diagnostic suggestions for doctors, help identify the severity and type of TBI, but also predict mental state levels. For example, Dabek et al. (2021) studied the potential of various machine learning techniques in predicting the development of psychological issues in patients with first-time mild traumatic brain injury (mTBI). The study found that these technologies could predict mental health risks based on patient data with high accuracy, providing a promising tool for early identification and intervention of psychological problems post-mTBI.

Decision support: By analyzing patient historical data, machine learning can predict the effectiveness of specific treatment plans, helping doctors choose the most appropriate treatment methods. For example, Moyer et al. (2022) developed a predictive model using machine learning techniques that accurately predicts whether moderate-to-severe traumatic brain injury (TBI) patients will require emergency neurosurgery within 24 h post-injury. The model uses key clinical factors for prediction, providing doctors with a rapid decision-making tool to improve patient outcomes (Moyer et al., 2022).

### 3.3 Real-time monitoring and risk assessment

During TBI treatment, patients’ conditions can change rapidly, making real-time monitoring and risk assessment crucial. Machine learning can analyze multimodal monitoring data in real time to identify early signals of condition changes and guide dynamic adjustments to treatment plans.

Dynamic monitoring systems: Through real-time data flow analysis, machine learning models can continuously monitor patients’ physiological states and automatically detect potential danger signals. For instance, when monitoring ICP, models can identify patterns of abnormal intracranial pressure elevation and alert doctors to take intervention measures. Ye et al. (2022) proposed a machine learning-based method for continuously predicting intracranial pressure changes in traumatic brain injury patients. By analyzing clinical data and training models, the study showed that this method could accurately predict short- and long-term changes in intracranial pressure, providing a new tool for non-invasive intracranial pressure monitoring and improving clinical management. The model’s overall performance includes an average accuracy of 94.62%, sensitivity of 74.91%, specificity of 94.83%, and a root mean square error of approximately 2.18 mmHg (Ye et al., 2022).

Risk assessment tools: Machine learning models can identify risk factors associated with complications and mortality by analyzing patients’ multimodal data. For example, Amorim et al. (2019) developed a model predicting early TBI mortality in low- and middle-income countries (LMIC) using machine learning methods. This model predicts early mortality risk based on key clinical features, providing a tool for early identification of high-risk TBI patients in LMIC regions and improving treatment and survival rates (Amorim et al., 2019).

During TTM, it is important to address the potential trade-offs between ICP and PbtO2. In some cases, PbtO2 (brain tissue oxygen pressure) may decrease during cooling, particularly when ventilation settings are suboptimal (e.g., latent over-ventilation). This phenomenon can result in hypoxia despite maintaining ICP control, and thus, balancing both ICP and PbtO2 becomes critical for effective neuroprotection. To guide clinicians, the following 4-step control heuristic can be proposed:

1. Prioritize ICP control: Keep ICP below a specified threshold (e.g., ICP < 20 mmHg) while maintaining PbtO2 within the target range (e.g., PbtO2 ≥ 20 mmHg).

2. Adjust ventilation if PbtO2 drops: If PbtO2 decreases below the target, first consider adjusting ventilation parameters (e.g., PaCO2 or FiO2) to optimize brain oxygenation, before raising body temperature.

3. Guardrails for shivering: If shivering is detected, consider sedation or muscle relaxants to prevent shivering induced ICP spikes and ensure the target temperature range is maintained.

4. Rewarming considerations: During the rewarming phase, carefully monitor both ICP and PbtO2. Rebound ICP is a common concern, and rewarming rates should be personalized, ideally predicted by machine learning models that take into account patient-specific physiological data.

Machine learning can significantly aid in this process by predicting the risk of PbtO2 drops or ICP elevation based on real-time monitoring data. For example, a machine learning model could analyze ICP, PbtO2, EtCO2, and other critical variables to forecast potential crises, helping clinicians to take proactive actions such as adjusting ventilation or cooling rates. By integrating multiple monitoring modalities, machine learning models can offer personalized recommendations, optimizing both ICP control and PbtO2 maintenance, and improving the safety and efficacy of TTM.

### 3.4 Personalized medicine and prognosis prediction

Personalized medicine is the future direction of TBI management (Savulich et al., 2018), with machine learning showing enormous potential in this area. By analyzing patient-specific physiological and pathological features, machine learning can tailor treatment and management plans for each patient.

Personalized treatment plan development: Machine learning can analyze multimodal monitoring data to identify different patients’ response patterns to treatments, thus creating personalized treatment plans for precise treatment and discharge planning (Azad et al., 2022). For instance, Satyadev et al. (2022) successfully developed a model predicting the discharge destination of traumatic brain injury patients using machine learning techniques. The model identified key factors affecting discharge outcomes from clinical data and showed high predictive accuracy, aiding healthcare professionals in making more precise decisions for patient rehabilitation paths and resource allocation (Satyadev et al., 2022). Their study developed a machine learning model to predict three-class discharge disposition in TBI patients (*n* = 5,292) using 84 features, including vital signs, demographics, and injury details. The random forest model demonstrated the best performance, with a weighted average area under the receiver operating characteristic curve (AUC) of 0.84 (95% CI 0.81–0.87) and a weighted average precision-recall AUC of 0.85 (95% CI 0.82–0.88). These models can improve patient triage and treatment, particularly for mild and moderate TBI cases.

Prognosis prediction models: By combining historical and multimodal monitoring data, machine learning models can predict patients’ long-term prognosis and recovery progress. This not only helps doctors assess treatment effectiveness but also provides more accurate prognosis information for patients and their families. Courville et al. (2023) conducted a systematic review and meta-analysis to assess the performance and accuracy of various machine learning algorithms in predicting TBI patient clinical outcomes. Despite limitations such as sample size and data quality, these algorithms generally showed potential in predicting TBI outcomes, providing valuable decision support tools for medical decisions (Courville et al., 2023).

To provide a clearer overview of the performance of different machine learning approaches applied in TBI management, we further summarized key models, their data modalities, validation methods, and performance metrics in Table 3. This comparison highlights both the potential and the limitations of each model, emphasizing the heterogeneity of current evidence and the necessity of rigorous external validation before clinical deployment.

**TABLE 3:** Comparative performance of machine learning models applied in traumatic brain injury (TBI) management.

Model	Data modality	Task	Validation	Performance	Key limitations
CNN	EEG	Seizure detection, consciousness prediction	Internal cross-validation	AUC≈0.81; Accuracy≈80%	Sensitive to noise; requires large datasets
RNN	EEG (continuous)	Epileptiform abnormality detection	External validation	Accuracy≈80.8%	Risk of overfitting; interpretability challenges
Random forest	ICP + Clinical	ICP prediction, crisis detection	Multi-site external validation	RMSE≈2.18 mmHg; Accuracy≈94%; Sensitivity≈75%	Data drift across sites; artifact sensitivity
SVM	CBF (LDF/NIRS)	Ischemia detection	Internal CV; temporal split	AUC≈0.75; Sensitivity≈70%	Small sample sizes; limited imaging resolution
Logistic regression	PbtO2	Prognosis, oxygenation prediction	External validation	AUC≈0.72; PPV moderate	Calibration issues; poor external generalization
U-Net/CNN-based	CT/MRI imaging	Lesion segmentation, severity classification	Multi-site external validation	Dice≈0.85; IoU≈0.80	Limited generalizability; time-consuming training

## 4 Hypothermic neuroprotection and other treatment strategies

**FIGURE 3:**
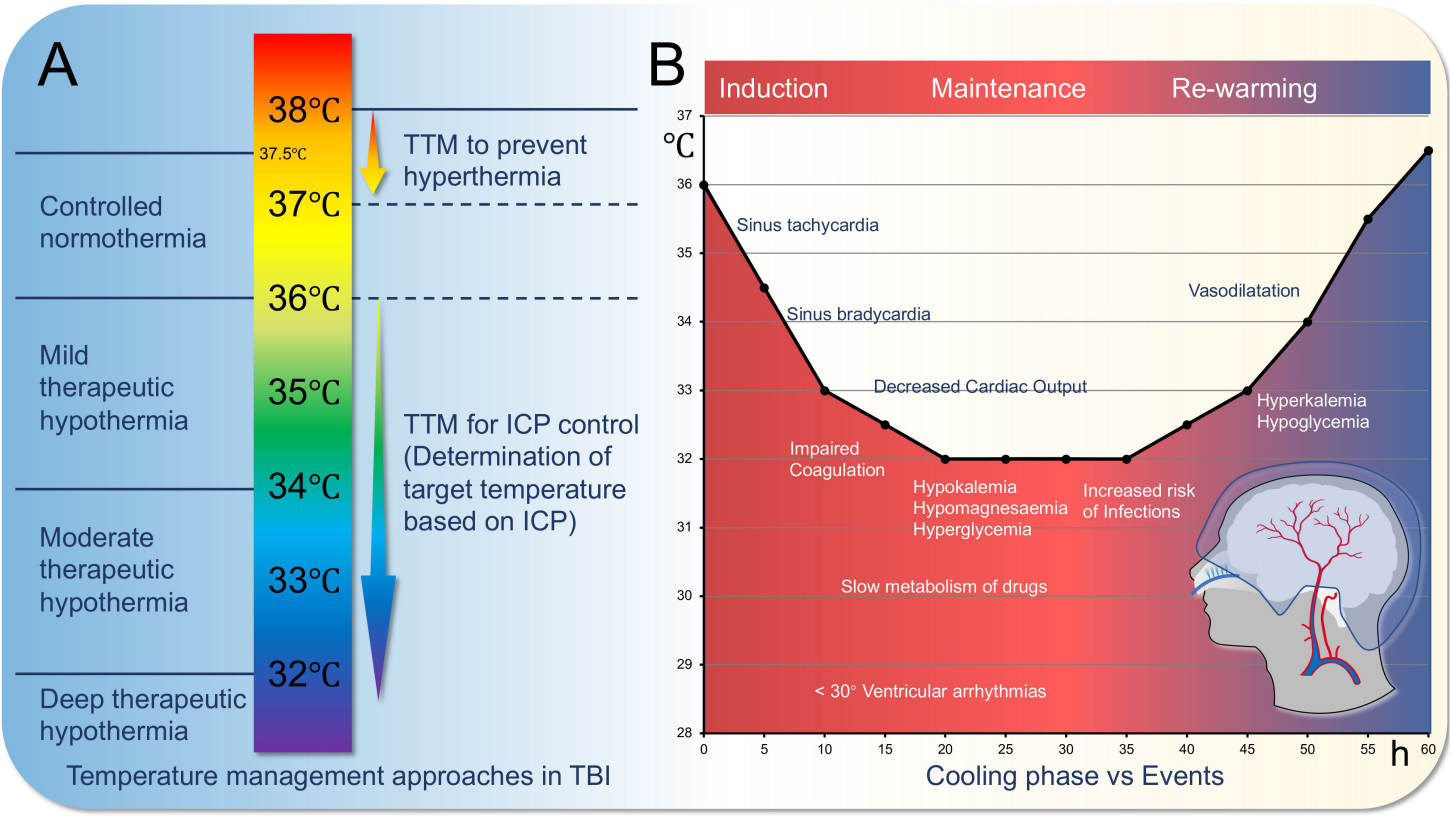
Targeted temperature management for traumatic brain injury (TBI). **(A)** Variations in the approach to temperature management in severe traumatic brain injury. Conventional therapeutic hypothermia is depicted on the left, while the concept of therapeutic hypotherm (TTM) for severe TBI is illustrated on the right. **(B)**. Cooling phase vs. events.

Targeted Temperature Management (TTM), also known as Therapeutic Hypothermia (TH), is a cornerstone of neuroprotective strategies. Hypothermic neuroprotection and other treatment strategies play a crucial role in TBI management by improving patient outcomes and reducing mortality and morbidity. These strategies include hypothermic neuroprotection, pharmacological treatments, and rehabilitation training, which will be discussed in detail below along with their potential integration with machine learning technologies (Andresen et al., 2015; Hong et al., 2022; Kawakita et al., 2024) (as shown in Figure 3).

### 4.1 Basic concepts and processes of hypothermic neuroprotection

Hypothermic neuroprotection, as a treatment strategy with significant neuroprotective effects, has gained widespread attention in TBI management. Its basic principle is to reduce the patient’s body temperature to slow down metabolic processes after brain injury, thereby reducing energy consumption and oxidative stress in brain cells (Parranto et al., 2025): lowering metabolic rate and restoring oxygen supply-demand balance. It reduces excitotoxicity, limits inflammation, prevents ATP depletion, reduces free radical production, and prevents intracellular calcium overload, avoiding apoptosis. Methods include external cooling methods such as ice blankets, ice packs, alcohol baths, cold water immersion, cold saline gastric lavage, helmet devices, and internal methods such as infusion of cold saline via central venous catheter or direct reduction of blood temperature (Andresen et al., 2015). Cooling treatment parameters should be adjusted according to the patient’s GCS (Glasgow Coma Scale) score. For patients with a GCS score of 3–5, a faster cooling rate may be required to minimize neural damage, while patients with higher GCS scores (e.g., 6–8) may not need as aggressive cooling treatment. The target temperature should be maintained between 32 °C and 35 °C, but the specific temperature range and duration of treatment should be further personalized based on the patient’s physiological state.

In TBI, hypothermic treatment can reduce brain edema, inhibit inflammatory responses, and decrease free radical production, all of which contribute to neurofunctional recovery. Hypothermic treatment is mainly divided into induction phase, maintenance phase, and rewarming phase, with physiological changes occurring at each stage (Jo, 2022). Additionally, it is worth noting that for severe traumatic brain injury (sTBI) patients, therapeutic hypothermia may require extended treatment time (Hui et al., 2021).

### 4.2 Expert consensus on hypothermic neuroprotection

In 2024, the ESICM/NACCS (European Society of Intensive Care Medicine/Neurocritical Care Society) consensus on best practices for targeted temperature management post-TBI provides guidelines for managing patients to optimize outcomes (Lavinio et al., 2024). Key recommendations include: (1). Indications: Initiate targeted temperature management (TTM) for severe TBI patients (Glasgow Coma Scale score ≤ 8) who do not comply with post-resuscitation orders. (2). Target Temperature: Maintain 32 °C and 35 °C (89.6–95°F) for at least 48 h to minimize secondary brain injury and improve neurological outcomes. (3). Initiation and Duration: Start TTM within 6 h of injury; duration should be personalized based on clinical response and evolving brain injury. (4). Methods: Use surface cooling devices, intravascular cooling catheters, or other methods to achieve and maintain target temperature. (5). Monitoring and Complications: Continuously monitor temperature and neurological status; manage complications such as hypotension, electrolyte imbalances, infections, and coagulopathy during TTM. (6). Multimodal Approach: TTM should be part of a multimodal approach to managing severe TBI, which may include surgical interventions, pharmacological treatments, and rehabilitation strategies tailored to individual patient needs. These consensus recommendations aim to standardize and improve the treatment and care of severe TBI patients by providing evidence-based guidelines for targeted temperature control, potentially improving outcomes and reducing morbidity associated with secondary brain injury.

The following Table 4 summarizes the use of therapeutic hypothermia across different TBI populations, outlining the target temperature range, timing and duration of cooling, primary outcomes, complications, and the net signal of the treatment’s effectiveness. It also highlights key caveats associated with each treatment approach, offering insights into the complexities of managing TBI with hypothermic neuroprotection. By reviewing the data from various studies on severe and mixed TBI, the table helps contextualize the varying responses to therapeutic hypothermia, guiding personalized treatment strategies for optimal patient care.

**TABLE 4:** Overview of therapeutic hypothermia parameters and outcomes in traumatic brain injury (TBI).

Population	Indication	Target temp/range	Timing/duration	Primary outcomes	Complications	Net signal	Key caveats
Severe TBI (sTBI)	ICP control	32 °C–34 °C	Induction within 6 h, maintain for 48 h	Reduced ICP, improved survival rate	Hypotension, infection, electrolyte imbalances	Benefit	Variability in ICP response, risk of rewarming rebound ICP
Mixed TBI (sTBI + mTBI)	Neuroprotection	33 °C–35 °C	Induction within 4 h, maintain for 72 h	Neuroprotective effects, decreased mortality	Coagulopathy, arrhythmias, shivering	Neutral	Population heterogeneity, timing uncertainty
Severe TBI (sTBI)	Neuroprotection + ICP control	32 °C–35 °C	Induction within 6 h, maintain for 48–72 h	Cognitive recovery, decreased secondary brain injury	Infection risk, cardiovascular instability	Neutral	Lack of long-term cognitive data, variable patient responses
Mixed TBI	Neuroprotection (preclinical)	33 °C–34 °C	Variable, depending on patient response	Inflammation reduction, brain edema control	Shivering, Sedation complications	Harm (inconsistent outcomes)	Unclear optimal treatment duration, lack of personalized monitoring
Severe TBI (sTBI)	Neuroprotection + ICP control	30 °C–32 °C	Induction within 4 h, maintain for 72 h	Reduced brain damage, improved functional outcomes	Hypothermic complications, coagulopathy	Benefit	Uncertainty regarding optimal rewarming phase

### 4.3 Necessity and potential approaches of machine learning in hypothermic brain protection

In clinical applications, hypothermic brain protection faces challenges such as determining the optimal cooling timing, temperature range, and duration. These parameters may vary among patients, necessitating personalized implementation of hypothermia therapy. For instance, Targeted Temperature Management (TTM) combined with brain tissue oxygen pressure (PbtO_2_) monitoring plays a crucial role in TBI treatment. TTM may reduce intracranial pressure by lowering body temperature, but its effect on PbtO_2_ can vary between patients, sometimes even causing a decrease in PbtO_2_, possibly related to latent overventilation during cooling. Therefore, TTM and PbtO_2_ monitoring need to be personalized and adjusted based on the patient’s specific condition and risk assessment (Cujkevic-Plecko et al., 2023). Machine learning can play a significant role in this process by analyzing large amounts of multimodal monitoring data to identify personalized optimal treatment parameters. Additionally, hypothermia therapy may lead to complications such as increased infection risk and arrhythmias, so close monitoring of the patient’s physiological status is essential. Machine learning can identify potential risks early through real-time data analysis, guiding clinical intervention decisions to improve the safety and efficacy of hypothermic brain protection. Machine learning can optimize hypothermic treatment parameters by analyzing multimodal monitoring data such as ICP (intracranial pressure) and PbtO2 (brain tissue oxygen pressure). For instance, when ICP exceeds a certain threshold, machine learning models can suggest increasing the cooling rate or adjusting the target temperature based on changes in PbtO2 to ensure adequate oxygenation of brain tissue.

Machine learning applications in brain injury hypothermia therapy are an emerging and promising field. Although still in the research stage, it has shown some potential applications and advantages:

1. Prediction and personalization of treatment: Machine learning can analyze large datasets, including clinical, imaging, and physiological data, to predict a patient’s response to hypothermia therapy. This helps doctors create personalized treatment plans to improve outcomes.
2. Optimization of treatment strategies: By analyzing patient data under different treatment strategies, machine learning can help determine the best hypothermia parameters (e.g., cooling rate, target temperature), thus optimizing the treatment plan and improving success rates.
3. Prediction of complications and patient outcomes: Machine learning can identify risks of complications and predict long-term outcomes such as survival rates and neurological recovery.
4. Imaging analysis support: Machine learning has potential in analyzing brain imaging data. It can automatically identify types, locations, and severity of brain injuries, providing more accurate diagnostic and treatment recommendations.
5. Real-time monitoring and feedback: Combining real-time monitoring technology with machine learning can analyze physiological parameters and feedback data, helping adjust the implementation of hypothermia therapy to ensure efficacy and patient safety.

Despite its early stage and need for extensive clinical validation, machine learning shows promise in improving treatment strategies and personalized medicine. As technology and data accumulate, machine learning is expected to play a greater role in providing more effective treatment and care solutions for patients.

The following Table 5 summarizes key aspects of the validation process, data handling methods, performance stability, and clinical utility for various machine learning models applied to TBI monitoring, including EEG, ICP, CBF, PbtO2, and imaging. It provides an overview of the validation techniques used, such as cross-validation, temporal splits, and multi-site external validation, as well as how missing data and potential data leakage were addressed. Additionally, the table highlights the performance stability and clinical utility of these models, shedding light on their current state and limitations for practical application in TBI management.

**TABLE 5:** Performance evaluation and validation of machine learning models in traumatic brain injury (TBI) monitoring.

Study type	Validation type	Data leakage checks	Missing-data handling	Performance stability	Calibration/clinical utility
Machine learning models for EEG	Internal CV, temporal split, external Validation	Leakage not reported	Imputation techniques used (mean imputation, regression)	Moderate stability, Further validation required	Calibration reported in internal studies, not external
Machine learning models for ICP	Multi-site external validation	Leakage checks not performed	Imputation with regression techniques	High stability with slight drift over time	Calibration not reported, potential for clinical utility uncertain
Machine learning models for CBF	Internal CV, external validation	Leakage checks reported	Missing data handled via multiple imputation	Stable performance with limited data variance	No clinical utility or decision-curve analysis
Machine learning models for PbtO2	Temporal split, Cross-validation, external validation	Leakage checks not reported	Missing data handled by mean imputation and regression	Performance stable with small sample size	No calibration, Clinical utility unclear
Machine learning models for imaging	Cross-validation, Multi-site validation	Leakage checks reported	Missing data handled via deletion methods	Stability issues, Variance across sites	Calibration and clinical utility reported for most models

### 4.4 Clinical uncertainties and challenges in the application of hypothermic therapy for traumatic brain injury

#### 4.4.1 Theoretical basis of hypothermic therapy vs. clinical uncertainties

Theoretical basis of hypothermic therapy: Hypothermic therapy (Targeted Temperature Management, TTM) is based on the principle of reducing brain temperature to slow down metabolic processes, thereby reducing energy consumption and oxidative stress. The main mechanisms are:

1. Metabolic Suppression: By lowering body temperature, the metabolic rate of brain cells is slowed, reducing energy consumption and preventing cell death and tissue necrosis due to energy deficiency.

2. Reduction of ICP (Intracranial Pressure): Lowering the temperature may help reduce brain edema, thus lowering ICP. This can alleviate secondary brain injury caused by elevated ICP by reducing cerebral blood flow.

3. Suppression of Inflammatory Response: Cooling reduces the activity of the immune system, inhibiting inflammation and minimizing neuronal damage caused by inflammatory cell infiltration.

Clinical uncertainties and challenges: Despite the promising theoretical benefits, the clinical application of TTM faces several uncertainties and challenges:

1. Heterogeneity of patient populations: The effectiveness of TTM may vary significantly between different patients. For instance, patients with severe traumatic brain injury (sTBI) may respond differently compared to those with mild traumatic brain injury (mTBI). Age, comorbidities, and other factors contribute to these differences.

2. Timing of treatment: The optimal timing to initiate TTM is still unclear. Research suggests that earlier initiation of cooling may provide better outcomes, but determining the exact window for treatment remains challenging.

3. Rewarming risks: The rewarming phase (the process of gradually raising the body temperature) poses a risk of ICP rebound. If rewarming is not controlled carefully, it could lead to an increase in ICP, exacerbating brain injury.

4. Infection and coagulopathy: Hypothermic therapy may suppress the immune system, increasing the risk of infections. Additionally, hypothermia can impair coagulation, raising the risk of bleeding, which complicates the treatment further.

#### 4.4.2 Rewarming process and ICP rebound analysis

ICP rebound during rewarming: The rewarming process is critical in TTM. While hypothermia helps reduce brain edema and control ICP, it may lead to ICP rebound during the rewarming phase. This rebound could be due to changes in cerebral blood flow and blood-brain barrier permeability as the brain temperature rises. Studies have shown that rewarming too quickly can lead to a sudden increase in ICP, which may further impair brain function and increase secondary injury.

It is essential to control the rewarming rate precisely to avoid a rapid rise in ICP. Research suggests that the ideal rewarming rate should be slow and controlled to minimize ICP fluctuations.

Machine learning’s role in predicting safe rewarming trajectories: Machine learning (ML) could be pivotal in predicting safe rewarming trajectories by analyzing multimodal data from various monitoring systems (such as ICP, PbtO2, EtCO2, MAP, and ventilation settings). By integrating data from these different sources, ML models could help predict potential ICP spikes during rewarming and recommend adjustments to minimize risks.

1. Multimodal Data Integration: ML models can integrate real-time data from EEG, ICP, PbtO2, and other monitoring systems to create a comprehensive patient status model. This could forecast the risk of ICP increase and provide recommendations for adjusting treatment strategies.

2. Personalized Rewarming Strategies: ML could enable the development of personalized rewarming plans for each patient based on their individual response to TTM. For example, if ICP is approaching a critical threshold, the model could suggest adjusting ventilation parameters (e.g., PaCO2 or FiO2) or delay rewarming to ensure safety.

3. Real-Time Monitoring: By continuously monitoring multiple physiological parameters, ML models can detect early signs of ICP rebound and prompt timely interventions, such as adjusting sedation levels or ventilation settings to maintain ICP within a safe range.

### 4.4 Other treatment strategies

Machine learning plays a crucial role in optimizing treatment strategies for TBI, particularly in drug therapy and rehabilitation training. By analyzing large amounts of patient data, machine learning can facilitate the personalization of treatment plans, optimizing their effectiveness.

#### 4.4.1 Personalized drug dosage prediction

Drug therapy is another crucial component in TBI management, aiming to mitigate secondary pathological processes following brain injury. Commonly used drugs include antioxidants, anti-inflammatory agents, and neuroprotective agents. Antioxidants neutralize free radicals generated during injury, reducing oxidative stress on neurons (Fesharaki-Zadeh, 2022). Anti-inflammatory agents reduce brain edema and inflammation by inhibiting the release of inflammatory mediators (Kalra et al., 2022; Lu et al., 2025). Neuroprotective agents such as creatine (Newman et al., 2023) and adenosine (Bozdemir et al., 2021) help maintain cell membrane integrity and prevent neuronal apoptosis (Tang et al., 2023; Zamanian et al., 2022). Medications may vary based on the disease stage: during the acute phase, tranexamic acid, antiepileptic drugs, hyperosmotic agents, and anesthetics are primary treatments and have proven effective. In later stages, SSRIs, SNRIs, antipsychotics, zolpidem, amantadine, and other drugs are used for neuropsychological issues, while muscle relaxants and botulinum toxin are used for spasticity (Tani et al., 2022).

Although drug therapy shows efficacy in TBI, its use requires careful consideration of individual factors. Machine learning can help optimize drug therapy (Lipponen et al., 2019) by analyzing patient data to predict responses to specific drugs, enabling precision medicine. Moreover, machine learning models can identify potential side effects and guide physicians in risk assessment and treatment adjustments (Fucich et al., 2020). Suppose a severe TBI patient with a GCS score of 5 has an initial ICP of 25 mmHg and a PbtO2 of 12 mmHg. Based on this data, the machine learning model can predict that the cooling rate should be set to a decrease of 0.5 °C per hour, and the target temperature should be adjusted to 33 °C for a duration of 48 hours, to ensure optimal neuroprotective effects.

Machine learning can predict the optimal drug dosage based on a patient’s genotype, particularly genes related to drug metabolism. For example, the metabolism rates of antiepileptic drugs such as phenytoin and carbamazepine are influenced by variations in the CYP450 gene family. By establishing machine learning models, it is possible to predict the most appropriate drug dosage based on a patient’s genetic data, drug concentration, and clinical symptoms, thereby optimizing therapeutic effects. With this personalized prediction, doctors can more accurately adjust drug dosages, minimizing the risks of overdose or insufficient dosage.

#### 4.4.2 Dynamic adjustment of rehabilitation plans

Rehabilitation training is an essential component of recovery for TBI patients, aimed at promoting functional recovery and improving long-term prognosis (Bayley et al., 2023). Physical rehabilitation includes exercise training, balance training, and functional training to enhance motor abilities and daily living functions (Gmelig Meyling et al., 2022). Cognitive rehabilitation addresses common cognitive impairments in TBI patients, such as memory, attention, and executive function deficits, through specialized training to improve cognitive function (Paggetti et al., 2025). Additionally, psychological support is crucial in TBI recovery, helping patients cope with emotional disorders and psychological stress through interventions and supportive therapy, promoting holistic recovery (Howlett et al., 2022). Machine learning can analyze rehabilitation training data, assess training effectiveness, and recommend personalized rehabilitation plans (Appiah Balaji et al., 2023). By tracking patient progress, machine learning models can adjust training plans in real-time to ensure each patient receives the most appropriate rehabilitation program, thereby improving recovery and quality of life.

In rehabilitation training, machine learning can dynamically adjust the intensity and frequency of exercises by analyzing large amounts of data from motion sensors (such as accelerometers and gyroscopes). For instance, machine learning models can monitor patients’ motion data in real-time (e.g., gait, limb movement amplitude) to assess rehabilitation progress. Based on changes in motor abilities, the training plan can be automatically adjusted. By making these dynamic adjustments, machine learning helps optimize the rehabilitation process, preventing overtraining or insufficient training.

#### 4.4.3 Example of machine learning in drug dosage and rehabilitation training

Drug dosage optimization: Take the antiepileptic drug phenytoin as an example. Machine learning can establish personalized drug dosage prediction models using patient genotype data (e.g., CYP450 gene variations), blood drug concentrations, and clinical symptoms. For instance, patients with CYP2C9 gene variants may require lower doses of phenytoin, and machine learning can automatically optimize the dosage based on this information.

Rehabilitation training adjustment: Using motion sensors (such as smart gloves) to monitor hand movements in TBI patients, machine learning models can adjust the intensity and duration of rehabilitation training based on movement amplitude, frequency, and training progress. If the patient’s hand movements recover more slowly, the model can automatically increase the training intensity; conversely, if the patient feels fatigued or limited in movement, the model can reduce the training load accordingly.

**FIGURE 4:**
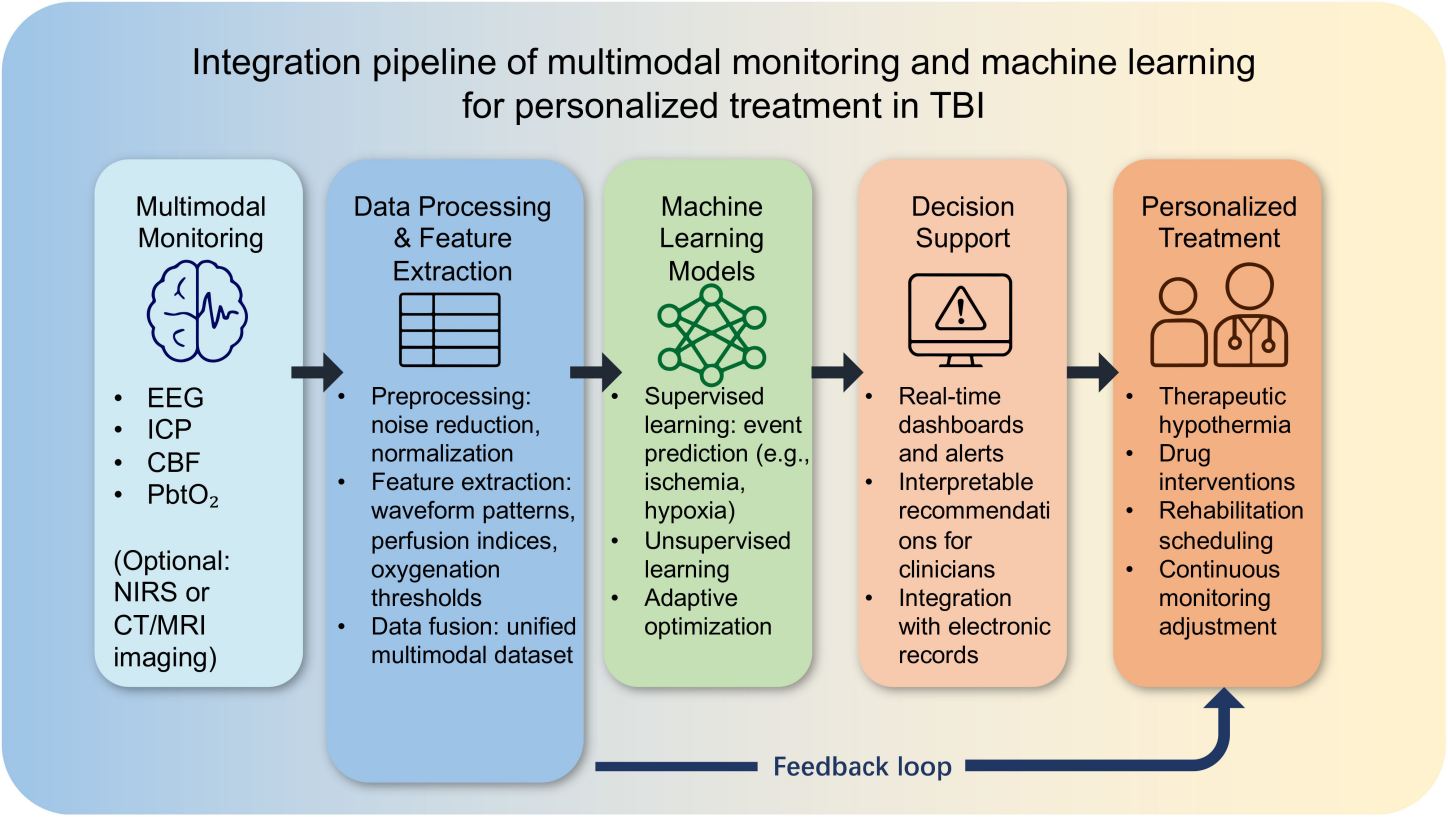
Integration pipeline of multimodal monitoring and machine learning for personalized treatment in traumatic brain injury (TBI).

To illustrate the practical implementation of multimodal monitoring combined with machine learning in TBI management, we present an integration pipeline in Figure 4. This schematic demonstrates how raw physiological data from various monitoring modalities—such as EEG, intracranial pressure (ICP), cerebral blood flow (CBF), and brain tissue oxygenation (PbtO2)—are first preprocessed, followed by feature extraction and data fusion. Machine learning models then integrate these processed data to enable event prediction, patient stratification, and adaptive optimization of treatment strategies. The outputs are delivered through a decision-support system, providing clinicians with guidance for personalized interventions. Importantly, a feedback loop facilitates continuous refinement, ensuring that treatment personalization adapts dynamically to each patient’s evolving condition.

The following Table 6 outlines the optimal cooling parameters, including target temperature, cooling rate, and maintenance duration, based on the patient’s GCS score. It also highlights the correlation between these parameters and the 6-month Glasgow Outcome Scale (GOS) score, reflecting their potential impact on recovery. This personalized approach aims to improve the efficacy of hypothermic therapy for TBI patients.

**TABLE 6:** Personalized cooling parameters based on GCS score and recovery outcomes in traumatic brain injury (TBI) patients.

Treatment parameter	GCS score	Optimal temperature	Cooling rate	Maintenance duration	6-month GOS score correlation
Target temperature	3–5	32 °C–33 °C	0.5 °C/h	48 h	High correlation with good recovery
	6–8	33 °C–34 °C	0.2 °C/h	24 h	Moderate correlation with recovery
Cooling rate	3–5	32 °C	0.5 °C/h	48 h	Strong impact on recovery
	6–8	34 °C	0.3 °C/h	24 h	Moderate impact

## 5 Discussion

### 5.1 Current evidence and principal limitations

The integration of multimodal bedside monitoring with ML has emerged as a promising approach to refine TBI management. By leveraging physiological signals such as EEG, ICP, CBF, and PbtO2, ML models have demonstrated potential in detecting seizures, forecasting ICP crises, stratifying risk, and guiding therapeutic interventions including TTM. For instance, recurrent neural networks trained on continuous EEG have achieved accuracies of ∼80% in detecting epileptiform discharges, while deep learning–based approaches for short-term ICP prediction have reported clinically meaningful error margins.

Nevertheless, enthusiasm must be tempered by several limitations. First, evidence from hypothermia trials illustrates the challenges of translating promising physiological mechanisms into consistent clinical benefit. Large-scale RCTs such as Eurotherm3235 and POLAR failed to establish uniform improvements in long-term functional outcomes, suggesting that non-stratified cooling strategies may obscure subgroup-specific benefits. Second, the heterogeneity of multimodal data—arising from differences in acquisition devices, protocols, and patient populations—complicates model generalizability. Small sample sizes further increase the risk of overfitting, with many studies still limited to retrospective, single-center cohorts. Finally, interpretability remains a key barrier: while deep learning can capture non-linear interactions, its “black-box” nature undermines clinical trust, especially for high-stakes interventions. More transparent methods such as logistic regression offer clarity but lack the capacity to model complex physiology. Together, these limitations underscore that while ML-enhanced multimodal monitoring is technically feasible, its clinical utility remains provisional.

### 5.2 Challenges in clinical translation

Moving from research to bedside deployment requires addressing methodological, operational, and regulatory challenges.

Validation hierarchy: A rigorous stepwise validation pathway is essential: internal cross-validation, temporal split validation, multi-site external validation, silent prospective deployment, and ultimately interventional RCTs. Yet, few published models progress beyond internal validation, raising concerns about reproducibility across centers.

Data governance and multi-center collaboration: FL offers a promising framework for training robust models without sharing raw patient data, thereby circumventing privacy barriers. However, its clinical adoption necessitates standardized data dictionaries, synchronized sampling frequencies, and harmonized labeling criteria across institutions.

Interpretability and human oversight: Clinical decision-support systems must output not only predictions but also interpretable rationales and uncertainty estimates. Approaches such as SHAP-based feature attribution and counterfactual explanations should be integrated as default outputs. When confidence is low, ML systems should trigger a human-in-the-loop fallback, ensuring that responsibility remains with the clinical team.

Safety and workflow integration: To avoid alert fatigue, models should provide prioritized, threshold-based outputs that align with existing clinical response pathways. Continuous performance monitoring and automatic recalibration are equally critical, given device drift and evolving patient populations.

Cost-effectiveness: Multimodal monitoring infrastructures and ML deployment impose substantial costs. Strategies should balance precision against feasibility, for example by developing lightweight models that rely on widely available variables (vital signs, basic ICP, or NIRS indices) in resource-limited settings.

### 5.3 Future directions and testable proposals

Future research must move beyond proof-of-concept toward rigorous clinical evaluation and real-world implementation. We propose the following priorities:

1. Adaptive, stratified clinical trials for ML-guided TTM. Future RCTs should employ adaptive or sequential multiple assignment randomized trial (SMART) designs, where ML algorithms stratify patients based on multimodal phenotypes and guide individualized cooling parameters. Hypotheses such as whether ML-optimized cooling rates improve ICP control without increasing infection risk can be explicitly tested.

2. Federated, multi-center model development. Large-scale federated learning initiatives should be established, enabling joint model training while preserving patient privacy. Such collaborations would mitigate site-specific biases and accelerate model generalizability.

3. Explainability and uncertainty quantification as regulatory standards. Clinical-grade ML systems must provide interpretable rationales and calibrated confidence intervals as part of routine outputs. For example, a recommendation to adjust temperature targets should be accompanied by explicit features driving the prediction (e.g., rising ICP trend, stable PbtO2, EEG desynchronization).

4. Model maintenance and drift monitoring. Post-deployment surveillance pipelines are needed to detect covariate drift, label drift, and performance degradation. Automatic recalibration and periodic retraining should be mandated to maintain safety and efficacy.

5. Lightweight models for resource-constrained settings. Developing simplified algorithms that rely on low-cost modalities such as vital signs and NIRS will expand accessibility, ensuring that ML benefits are not limited to high-resource centers.

6. Physiology-informed feature engineering. In addition to end-to-end deep learning, incorporating physiologically interpretable features—such as ICP pulse morphology or dynamic cerebrovascular reactivity indices—can both improve predictive accuracy and facilitate clinical acceptance.

7. Optimizing the role of NIRS. Evidence suggests that raw NIRS values alone provide limited prognostic power; however, derived indices such as cerebrovascular reactivity metrics may add value when integrated into multimodal ML models.

In summary, ML-enabled multimodal monitoring has demonstrated technical feasibility and early promise in TBI management, particularly in enhancing seizure detection, ICP prediction, and personalization of hypothermia protocols. Yet the field remains in an early translational stage, constrained by heterogeneous data, limited external validation, and interpretability challenges. Moving forward, the development of multicenter federated learning frameworks, stratified adaptive trial designs, and interpretable outputs with uncertainty quantification will be critical for bridging the gap between computational potential and bedside utility. Only through such interdisciplinary and methodologically rigorous efforts can ML evolve from experimental tool to a clinically transformative paradigm in TBI care.

## 6 Conclusion

Traumatic brain injury remains a heterogeneous and complex condition where conventional monitoring and treatment approaches often fail to capture individual variability. The integration of multimodal monitoring with machine learning provides a promising framework to address this challenge, offering opportunities for earlier event prediction, individualized therapeutic adjustment, and data-driven clinical decision support.

Current evidence demonstrates technical feasibility, with encouraging results in tasks such as seizure detection, ICP forecasting, and imaging-based lesion characterization. Yet translation into routine clinical care is limited by small sample sizes, insufficient external validation, and the lack of interpretability in complex models. Large-scale RCTs of hypothermia have further highlighted that “one-size-fits-all” strategies are unlikely to succeed, underscoring the need for stratified, personalized protocols.

Looking ahead, progress will depend on three key pillars: (i) building high-quality, multicenter datasets through federated learning and standardized protocols; (ii) embedding explainability, uncertainty quantification, and robust validation into all clinical-grade ML systems; and (iii) designing adaptive, phenotype-driven clinical trials to test whether ML-guided interventions can yield measurable improvements in outcomes.

In summary, ML-enabled multimodal monitoring represents an emerging paradigm in TBI care. While challenges in validation, interpretability, and deployment remain, the convergence of computational advances, collaborative data sharing, and rigorous clinical evaluation has the potential to transform TBI management from generalized protocols toward truly personalized, precision neurocritical care.

## References

[B1] Acosta J. Falcone G. Rajpurkar P. Topol E. (2022). Multimodal biomedical AI. *Nat. Med.* 28 1773–1784. 10.1038/s41591-022-01981-2 36109635

[B2] Amorim R. Oliveira L. Malbouisson L. Nagumo M. Simoes M. Miranda L. (2019). Prediction of early TBI mortality using a machine learning approach in a LMIC population. *Front. Neurol.* 10:1366. 10.3389/fneur.2019.01366 32038454 PMC6992595

[B3] Andresen M. Gazmuri J. Marín A. Regueira T. Rovegno M. (2015). Therapeutic hypothermia for acute brain injuries. *Scand. J. Trauma Resusc. Emerg. Med.* 23:42. 10.1186/s13049-015-0121-3 26043908 PMC4456795

[B4] Appavu B. Burrows B. Nickoles T. Boerwinkle V. Willyerd A. Gunnala V. (2021). Implementation of multimodality neurologic monitoring reporting in pediatric traumatic brain injury management. *Neurocrit. Care* 35 3–15. 10.1007/s12028-021-01190-8 33791948 PMC8012079

[B5] Appiah Balaji N. Beaulieu C. Bogner J. Ning X. (2023). Traumatic brain injury rehabilitation outcome prediction using machine learning methods. *Arch. Rehabil. Res. Clin. Transl.* 5:100295. 10.1016/j.arrct.2023.100295 38163039 PMC10757159

[B6] Ayasse T. Gaugain S. de Roquetaillade C. Hermans-Didier A. Kindermans M. Chousterman B. (2025). Association between cerebral oxygenation and usual parameters of cerebral perfusion in critically ill patients with acute brain injury. *J. Cereb. Blood Flow Metab.* 45 1059–1068. 10.1177/0271678X241310780 39763378 PMC11705312

[B7] Azad T. Shah P. Kim H. Stevens R. (2022). Endotypes and the path to precision in moderate and severe traumatic brain injury. *Neurocrit. Care* 37 259–266. 10.1007/s12028-022-01475-6 35314969

[B8] Bai Y. Lin Y. Ziemann U. (2021). Managing disorders of consciousness: The role of electroencephalography. *J. Neurol.* 268 4033–4065. 10.1007/s00415-020-10095-z 32915309 PMC8505374

[B9] Bartnik-Olson B. Alger J. Babikian T. Harris A. Holshouser B. Kirov I. (2021). The clinical utility of proton magnetic resonance spectroscopy in traumatic brain injury: Recommendations from the ENIGMA MRS working group. *Brain Imaging Behav.* 15 504–525. 10.1007/s11682-020-00330-6 32797399 PMC7882010

[B10] Barud M. Dabrowski W. Siwicka-Gieroba D. Robba C. Bielacz M. Badenes R. (2021). Usefulness of cerebral oximetry in TBI by NIRS. *J. Clin. Med.* 10:2938. 10.3390/jcm10132938 34209017 PMC8268432

[B11] Bayley M. Janzen S. Harnett A. Teasell R. Patsakos E. Marshall S. (2023). INCOG 2.0 guidelines for cognitive rehabilitation following traumatic brain injury: Methods, overview, and principles. *J. Head Trauma Rehabil.* 38 7–23. 10.1097/HTR.0000000000000838 36594856

[B12] Bischof G. Cross D. (2023). Brain trauma imaging. *J. Nucl. Med.* 64 20–29. 10.2967/jnumed.121.263293 36599475 PMC9841252

[B13] Bozdemir E. Vigil F. Chun S. Espinoza L. Bugay V. Khoury S. (2021). Neuroprotective roles of the adenosine A3 receptor agonist AST-004 in mouse model of traumatic brain injury. *Neurotherapeutics* 18 2707–2721. 10.1007/s13311-021-01113-7 34608616 PMC8804149

[B14] Cobianchi L. Piccolo D. Dal Mas F. Agnoletti V. Ansaloni L. Balch J. (2023). Surgeons’ perspectives on artificial intelligence to support clinical decision-making in trauma and emergency contexts: Results from an international survey. *World J. Emerg. Surg.* 18:1. 10.1186/s13017-022-00467-3 36597105 PMC9811693

[B15] Courville E. Kazim S. Vellek J. Tarawneh O. Stack J. Roster K. (2023). Machine learning algorithms for predicting outcomes of traumatic brain injury: A systematic review and meta-analysis. *Surg. Neurol. Int.* 14:262. 10.25259/SNI_312_2023 37560584 PMC10408617

[B16] Cujkevic-Plecko N. Rodriguez A. Anderson T. Rhodes J. (2023). Targeted temperature management and PbtO2 in traumatic brain injury. *Brain Spine* 3:102704. 10.1016/j.bas.2023.102704 38105803 PMC10724196

[B17] Dabek F. Hoover P. Jorgensen-Wagers K. Wu T. Caban J. (2021). Evaluation of machine learning techniques to predict the likelihood of mental health conditions following a first mTBI. *Front. Neurol.* 12:769819. 10.3389/fneur.2021.769819 35185749 PMC8847217

[B18] Dewan M. Rattani A. Gupta S. Baticulon R. Hung Y. Punchak M. (2019). Estimating the global incidence of traumatic brain injury. *J. Neurosurg.* 130 1080–1097. 10.3171/2017.10.JNS17352 29701556

[B19] Faghihpirayesh R. Ruf S. Rocca M. Garner R. Vespa P. Erdogmus D. (2021). Automatic detection of EEG epileptiform abnormalities in traumatic brain injury using deep learning. *Annu. Int. Conf. IEEE Eng. Med. Biol. Soc.* 2021 302–305. 10.1109/EMBC46164.2021.9630242 34891296 PMC8860400

[B20] Fernando S. Tran A. Cheng W. Rochwerg B. Taljaard M. Kyeremanteng K. (2019). Diagnosis of elevated intracranial pressure in critically ill adults: Systematic review and meta-analysis. *BMJ* 366:l4225. 10.1136/bmj.l4225 31340932 PMC6651068

[B21] Fesharaki-Zadeh A. (2022). Oxidative stress in traumatic brain injury. *Int. J. Mol. Sci.* 23:13000. 10.3390/ijms232113000 36361792 PMC9657447

[B22] Früh A. Sanchin A. Vajkoczy P. Wolf S. (2024). Bedside technique for the implantation of licox brain tissue oxygen probes in occipital regions: A technical note. *World Neurosurg.* 189 127–131. 10.1016/j.wneu.2024.06.008 38871288

[B23] Fucich E. Stielper Z. Cancienne H. Edwards S. Gilpin N. Molina P. (2020). Endocannabinoid degradation inhibitors ameliorate neuronal and synaptic alterations following traumatic brain injury. *J. Neurophysiol.* 123 707–717. 10.1152/jn.00570.2019 31913777 PMC7052644

[B24] Gmelig Meyling C. Verschuren O. Rentinck I. Engelbert R. Gorter J. (2022). Physical rehabilitation interventions in children with acquired brain injury: A scoping review. *Dev. Med. Child Neurol.* 64 40–48. 10.1111/dmcn.14997 34309829 PMC9292549

[B25] Gomez A. Froese L. Griesdale D. Thelin E. Raj R. van Iperenburg L. (2024). Prognostic value of near-infrared spectroscopy regional oxygen saturation and cerebrovascular reactivity index in acute traumatic neural injury: A CAnadian High-resolution traumatic brain injury (CAHR-TBI) cohort study. *Crit. Care* 28:78. 10.1186/s13054-024-04859-6 38486211 PMC10938687

[B26] Gomez A. Griesdale D. Froese L. Yang E. Thelin E. Raj R. (2023a). Temporal statistical relationship between regional cerebral oxygen saturation (rSO2) and brain tissue oxygen tension (PbtO2) in moderate-to-severe traumatic brain injury: A Canadian high resolution-TBI (CAHR-TBI) cohort Study. *Bioengineering* 10:1124. 10.3390/bioengineering10101124 37892854 PMC10604223

[B27] Gomez A. Sainbhi A. Stein K. Vakitbilir N. Froese L. Zeiler F. (2023b). Statistical properties of cerebral near infrared and intracranial pressure-based cerebrovascular reactivity metrics in moderate and severe neural injury: A machine learning and time-series analysis. *Intensive Care Med. Exp.* 11:57. 10.1186/s40635-023-00541-3 37635181 PMC10460757

[B28] Greener J. Kandathil S. Moffat L. Jones D. T. A. (2022). guide to machine learning for biologists. *Nat. Rev. Mol. Cell. Biol.* 23 40–55. 10.1038/s41580-021-00407-0 34518686

[B29] Hartings J. Andaluz N. Bullock M. Hinzman J. Mathern B. Pahl C. (2020). Prognostic value of spreading depolarizations in patients with severe traumatic brain injury. *JAMA Neurol.* 77 489–499. 10.1001/jamaneurol.2019.4476 31886870 PMC6990808

[B30] Hong J. Choi E. Park S. (2022). Selective brain cooling: A new horizon of neuroprotection. *Front. Neurol.* 13:873165. 10.3389/fneur.2022.873165 35795804 PMC9251464

[B31] Howlett J. Nelson L. Stein M. (2022). Mental health consequences of traumatic brain injury. *Biol. Psychiatry* 91 413–420. 10.1016/j.biopsych.2021.09.024 34893317 PMC8849136

[B32] Hu L. Yang S. Jin B. Wang C. (2022). Advanced neuroimaging role in traumatic brain injury: A narrative review. *Front. Neurosci.* 16:872609. 10.3389/fnins.2022.872609 35495065 PMC9043279

[B33] Hui J. Feng J. Tu Y. Zhang W. Zhong C. Liu M. (2021). Safety and efficacy of long-term mild hypothermia for severe traumatic brain injury with refractory intracranial hypertension (LTH-1): A multicenter randomized controlled trial. *EClinicalMedicine* 32:100732. 10.1016/j.eclinm.2021.100732 33681741 PMC7910713

[B34] Hwang S. Kwon N. Lee D. Kim J. Yang S. Youn I. (2025). A multimodal fatigue detection system using sEMG and IMU signals with a hybrid CNN-LSTM-attention model. *Sensors* 25:3309. 10.3390/s25113309 40968807 PMC12157230

[B35] Jo K. (2022). Target temperature management in traumatic brain injury with a focus on adverse events, recognition, and prevention. *Acute Crit. Care.* 37 483–490. 10.4266/acc.2022.01291 36442469 PMC9732187

[B36] Kalra S. Malik R. Singh G. Bhatia S. Al-Harrasi A. Mohan S. (2022). Pathogenesis and management of traumatic brain injury (TBI): Role of neuroinflammation and anti-inflammatory drugs. *Inflammopharmacology* 30 1153–1166. 10.1007/s10787-022-01017-8 35802283 PMC9293826

[B37] Kawakita K. Shishido H. Kuroda Y. (2024). Review of temperature management in traumatic brain injuries. *J. Clin. Med.* 13:2144. 10.3390/jcm13072144 38610909 PMC11012999

[B38] Kendall H. Van Kuijk S. Horst I. C. Dings J. T. Aries M. J. Haeren R. H. (2023). Difference between brain temperature and core temperature in severe traumatic brain injury: A systematic review. *J. Neurosurg. Sci.* 67 46–54. 10.23736/S0390-5616.21.05519-3 35301834

[B39] Lavinio A. Coles J. Robba C. Aries M. Bouzat P. Chean D. (2024). Targeted temperature control following traumatic brain injury: esicm/naccs best practice consensus recommendations. *Crit. Care* 28:170. 10.1186/s13054-024-04951-x 38769582 PMC11107011

[B40] Liang S. Ti Y. Li X. Zhou W. (2023). The protective role and mechanism of mild therapeutic hypothermia protection on brain cells. *Neuropsychiatr. Dis. Treat.* 19 1625–1631. 10.2147/NDT.S412227 37484118 PMC10361083

[B41] Ling X. Yang X. Wang P. Li Y. Wen Z. Wang J. (2025). Intratumoral and peritumoral heterogeneity based on CT to predict the pathological response after neoadjuvant chemoimmunotherapy in esophageal squamous cell carcinoma. *Int. J. Surg.* 10.1097/JS9.0000000000003422 Online ahead of print. 40968727 PMC12825770

[B42] Lins B. Anyaegbu C. Hellewell S. Papini M. McGonigle T. De Prato L. (2023). Cannabinoids in traumatic brain injury and related neuropathologies: Preclinical and clinical research on endogenous, plant-derived, and synthetic compounds. *J. Neuroinflammation* 20 77. 10.1186/s12974-023-02734-9 36935484 PMC10026409

[B43] Lipponen A. Natunen T. Hujo M. Ciszek R. Hämäläinen E. Tohka J. (2019). In vitro and in vivo pipeline for validation of disease-modifying effects of systems biology-derived network treatments for traumatic brain injury-lessons learned. *Int. J. Mol. Sci.* 20:5395. 10.3390/ijms20215395 31671916 PMC6861918

[B44] Lu J. Dai X. Xi S. Wang B. Zhang P. Fu X. (2025). Piperine protects against cerebral ischemic injury by regulating the Caspase-1-mediated pyroptosis pathway. *Front. Pharmacol.* 16:1601873. 10.3389/fphar.2025.1601873 40786035 PMC12332512

[B45] Maas A. Menon D. Manley G. Abrams M. Åkerlund C. Andelic N. (2022). Traumatic brain injury: Progress and challenges in prevention, clinical care, and research. *Lancet Neurol.* 21 1004–1060. 10.1016/S1474-4422(22)00309-X 36183712 PMC10427240

[B46] Mader M. Rotermund R. Lefering R. Westphal M. Maegele M. Czorlich P. (2021). The faster the better? Time to first CT scan after admission in moderate-to-severe traumatic brain injury and its association with mortality. *Neurosurg. Rev.* 44 2697–2706. 10.1007/s10143-020-01456-3 33340052 PMC8490239

[B47] Minta K. Brinkmalm G. Al Nimer F. Thelin E. Piehl F. Tullberg M. (2020). Dynamics of cerebrospinal fluid levels of matrix metalloproteinases in human traumatic brain injury. *Sci. Rep.* 10:18075. 10.1038/s41598-020-75233-z 33093584 PMC7582923

[B48] Moyer J. Lee P. Bernard C. Henry L. Lang E. Cook F. (2022). Machine learning-based prediction of emergency neurosurgery within 24 h after moderate to severe traumatic brain injury. *World J. Emerg. Surg.* 17:42. 10.1186/s13017-022-00449-5 35922831 PMC9351267

[B49] Muller J. Wang R. Milddleton D. Alizadeh M. Kang K. Hryczyk R. (2023). Machine learning-based classification of chronic traumatic brain injury using hybrid diffusion imaging. *Front. Neurosci.* 17:1182509. 10.3389/fnins.2023.1182509 37694125 PMC10484001

[B50] Newman J. Pekari T. Van Wyck D. (2023). Neuroprotection and therapeutic implications of creatine supplementation for brain injury complications. *Med. J.* 2023 31–38. 37042504

[B51] Paggetti A. Druda Y. Sciancalepore F. Della Gatta F. Ancidoni A. Locuratolo N. (2025). The efficacy of cognitive stimulation, cognitive training, and cognitive rehabilitation for people living with dementia: A systematic review and meta-analysis. *Geroscience* 47 409–444. 10.1007/s11357-024-01400-z 39485657 PMC11872969

[B52] Parranto M. Kung T. Liddle L. Khalid T. Thorkelsson A. Klahr A. (2025). A systematic review of depth-dependent cytoprotection with therapeutic hypothermia for cerebral ischemia. *Ther. Hypothermia Temp. Manag.* 10.1177/21537658251377958 Online ahead of print. 40956646

[B53] Parsa M. Rad H. Vaezi H. Hossein-Zadeh G. Setarehdan S. Rostami R. (2023). EEG-based classification of individuals with neuropsychiatric disorders using deep neural networks: A systematic review of current status and future directions. *Comput. Methods Programs Biomed.* 240:107683. 10.1016/j.cmpb.2023.107683 37406421

[B54] Pinggera D. Luger M. Bürgler I. Bauer M. Thomé C. Petr O. (2020). Safety of early MRI examinations in severe TBI: A test battery for proper patient selection. *Front. Neurol.* 11:219. 10.3389/fneur.2020.00219 32373042 PMC7179696

[B55] Pyrzowski J. Kałas M. Mazurkiewicz-Bełdzińska M. Siemiński M. (2024). EEG biomarkers for the prediction of post-traumatic epilepsy - A systematic review of an emerging field. *Seizure* 119 71–77. 10.1016/j.seizure.2024.05.006 38796954

[B56] Rohaut B. Calligaris C. Hermann B. Perez P. Faugeras F. Raimondo F. (2024). Multimodal assessment improves neuroprognosis performance in clinically unresponsive critical-care patients with brain injury. *Nat. Med.* 30 2349–2355. 10.1038/s41591-024-03019-1 38816609 PMC11333287

[B57] Roldán M. Abay T. Kyriacou P. (2020). Non-invasive techniques for multimodal monitoring in traumatic brain injury: Systematic review and meta-analysis. *J. Neurotrauma* 37 2445–2453. 10.1089/neu.2020.7266 32821023

[B58] Rot S. Dweek M. Gutowski P. Goelz L. Meier U. Lemcke J. (2020). Comparative investigation of different telemetric methods for measuring intracranial pressure: A prospective pilot study. *Fluids Barriers CNS* 17:63. 10.1186/s12987-020-00225-0 33069242 PMC7568395

[B59] Satyadev N. Warman P. Seas A. Kolls B. Haglund M. Fuller A. (2022). Machine learning for predicting discharge disposition after traumatic brain injury. *Neurosurgery* 90 768–774. 10.1227/neu.0000000000001911 35319523 PMC12273637

[B60] Savulich G. Menon D. Stamatakis E. Pickard J. Sahakian B. (2018). Personalised treatments for traumatic brain injury: Cognitive, emotional and motivational targets. *Psychol. Med.* 48 1397–1399. 10.1017/S0033291718000892 29636117

[B61] Schroder A. Lawrence T. Voets N. Garcia-Gonzalez D. Jones M. Peña J. (2021). A Machine learning enhanced mechanistic simulation framework for functional deficit prediction in TBI. *Front. Bioeng. Biotechnol.* 9:587082. 10.3389/fbioe.2021.587082 33748080 PMC7965982

[B62] Sconzo D. Wadhwa A. Balagurunath K. Berube M. Wetsel Z. Sakthiyendran N. (2025). Predictors of seizures in postoperative traumatic brain injury patients: A single center retrospective study. *Clin. Neurol. Neurosurg.* 257:109116. 10.1016/j.clineuro.2025.109116 40848611

[B63] Shang P. Zheng R. Wu K. Yuan C. Pan S. (2024). New insights on mechanisms and therapeutic targets of cerebral edema. *Curr. Neuropharmacol.* 22 2330–2352. 10.2174/1570159X22666240528160237 38808718 PMC11451312

[B64] Shao A. Zhu Z. Li L. Zhang S. Zhang J. (2019). Emerging therapeutic targets associated with the immune system in patients with intracerebral haemorrhage (ICH): From mechanisms to translation. *EBioMedicine* 45 615–623. 10.1016/j.ebiom.2019.06.012 31208948 PMC6642355

[B65] Tang Y. Liu Y. Zhou H. Lu H. Zhang Y. Hua J. (2023). Esketamine is neuroprotective against traumatic brain injury through its modulation of autophagy and oxidative stress via AMPK/mTOR-dependent TFEB nuclear translocation. *Exp. Neurol.* 366:114436. 10.1016/j.expneurol.2023.114436 37187276

[B66] Tani J. Wen Y. Hu C. Sung J. (2022). Current and potential pharmacologic therapies for traumatic brain injury. *Pharmaceuticals* 15:838. 10.3390/ph15070838 35890136 PMC9323622

[B67] Tas J. Czosnyka M. van der Horst I. Park S. van Heugten C. Sekhon M. (2022). Cerebral multimodality monitoring in adult neurocritical care patients with acute brain injury: A narrative review. *Front. Physiol.* 13:1071161. 10.3389/fphys.2022.1071161 36531179 PMC9751622

[B68] Tenovuo O. Diaz-Arrastia R. Goldstein L. Sharp D. van der Naalt J. Zasler N. (2021). Assessing the severity of traumatic brain injury-time for a change? *J. Clin. Med.* 10:148. 10.3390/jcm10010148 33406786 PMC7795933

[B69] Unadkat P. Rebeiz T. Ajmal E. De Souza V. Xia A. Jinu J. (2025). Neurobiological mechanisms underlying psychological dysfunction after brain injuries. *Cells* 14:74. 10.3390/cells14020074 39851502 PMC11763422

[B70] Vishwanath M. Jafarlou S. Shin I. Dutt N. Rahmani A. Jones C. (2021). Investigation of machine learning and deep learning approaches for detection of mild traumatic brain injury from human sleep electroencephalogram. *Annu. Int. Conf. IEEE Eng. Med. Biol. Soc.* 2021 6134–6137. 10.1109/EMBC46164.2021.9630423 34892516

[B71] VuE.BrownC.BradyK.HogueC. (2024). Monitoring of cerebral blood flow autoregulation: Physiologic basis, measurement, and clinical implications. *Br. J. Anaesth.* 132 1260–1273. 10.1016/j.bja.2024.01.043 38471987

[B72] Wang X. Chen S. Wang X. Song Z. Wang Z. Niu X. (2024). Application of artificial hibernation technology in acute brain injury. *Neural Regen. Res.* 19 1940–1946. 10.4103/1673-5374.390968 38227519 PMC11040302

[B73] Wu A. Yong Y. Pan Y. Zhang L. Wu J. Zhang Y. (2022). Targeting Nrf2-mediated oxidative stress response in traumatic brain injury: Therapeutic perspectives of phytochemicals. *Oxid. Med. Cell. Longev.* 2022:1015791. 10.1155/2022/1015791 35419162 PMC9001080

[B74] Wu X. Tao Y. Marsons L. Dee P. Yu D. Guan Y. (2021). The effectiveness of early prophylactic hypothermia in adult patients with traumatic brain injury: A systematic review and meta-analysis. *Aust. Crit. Care* 34 83–91. 10.1016/j.aucc.2020.05.005 32698987

[B75] Yan C. Mao J. Yao C. Liu Y. Yan H. Jin W. (2022). Neuroprotective effects of mild hypothermia against traumatic brain injury by the involvement of the Nrf2/ARE pathway. *Brain Behav.* 12:e2686. 10.1002/brb3.2686 35803901 PMC9392531

[B76] Ye G. Balasubramanian V. Li J. Kaya M. (2022). Machine learning-based continuous intracranial pressure prediction for traumatic injury patients. *IEEE J. Transl. Eng. Health Med.* 10:4901008. 10.1109/JTEHM.2022.3179874 35795876 PMC9252333

[B77] Yen T. Chang C. Chung C. Ko W. Yang C. Hsieh C. (2018). Neuroprotective effects of platonin, a therapeutic immunomodulating medicine, on traumatic brain injury in mice after controlled cortical impact. *Int. J. Mol. Sci.* 19:1100. 10.3390/ijms19041100 29642394 PMC5979356

[B78] Zamanian M. Taheri N. Opulencia M. Bokov D. Abdullaev S. Gholamrezapour M. (2022). Neuroprotective and anti-inflammatory effects of pioglitazone on traumatic brain injury. *Mediators Inflamm.* 2022:9860855. 10.1155/2022/9860855 35757108 PMC9232315

[B79] Zeiler F. Aries M. Czosnyka M. Smielewski P. (2022). Cerebral autoregulation monitoring in traumatic brain injury: An overview of recent advances in personalized medicine. *J. Neurotrauma* 39 1477–1494. 10.1089/neu.2022.0217 35793108

[B80] Zhang C. Ioachimescu A. (2025). Clinical manifestations and treatment of hypopituitarism due to traumatic brain injury. *Best Pract. Res. Clin. Endocrinol. Metab.* 39:101996. 10.1016/j.beem.2025.101996 40280796

[B81] Zhang K. Cai L. Song Y. Liu T. Zhao Y. (2021). Combining external medical knowledge for improving obstetric intelligent diagnosis: Model development and validation. *JMIR Med. Inform.* 9:e25304. 10.2196/25304 33970113 PMC8145091

[B82] Zhao X. Lu M. Yuan D. Xu D. Yao P. Ji W. (2019). Mitochondrial dysfunction in neural injury. *Front. Neurosci.* 13:30. 10.3389/fnins.2019.00030 30778282 PMC6369908

[B83] Zheng R. Lei Z. Yang R. Huang G. Zhang G. (2020). Identification and management of paroxysmal sympathetic hyperactivity after traumatic brain injury. *Front. Neurol.* 11:81. 10.3389/fneur.2020.00081 32161563 PMC7052349

